# Rapid and Robust Continuous Purification of High-Titer Hepatitis B Virus for In Vitro and In Vivo Applications

**DOI:** 10.3390/v13081503

**Published:** 2021-07-30

**Authors:** Jochen M. Wettengel, Bianca Linden, Knud Esser, Michael Laue, Benjamin J. Burwitz, Ulrike Protzer

**Affiliations:** 1 Institute of Virology, Technische Universität München/Helmholtz Zentrum München, 81675 Munich, Germany; Wettengel@tum.de (J.M.W.); Bianca.Linden@tum.de (B.L.); Knud.Esser@tum.de (K.E.); 2 Vaccine & Gene Therapy Institute, Oregon Health & Science University, Beaverton, OR 97006, USA; burwitz@ohsu.edu; 3 Advanced Light and Electron Microscopy (ZBS 4), Robert Koch Institute, 13353 Berlin, Germany; LaueM@rki.de

**Keywords:** hepatitis B virus, virus purification, heparin-affinity chromatography, sucrose-gradient ultracentrifugation, HBV high-titer virus stocks, HBV for in vitro and in vivo assays

## Abstract

Available treatments for hepatitis B can control the virus but are rarely curative. This led to a global initiative to design new curative therapies for the 257 million patients affected. Discovery and development of these new therapies is contingent upon functional in vitro and in vivo hepatitis B virus (HBV) infection models. However, low titer and impurity of conventional HBV stocks reduce significance of in vitro infections and moreover limit challenge doses in current in vivo models. Therefore, there is a critical need for a robust, simple and reproducible protocol to generate high-purity and high-titer infectious HBV stocks. Here, we outline a three-step protocol for continuous production of high-quality HBV stocks from supernatants of HBV-replicating cell lines. This purification process takes less than 6 h, yields to high-titer stocks (up to 1 × 10^11^ enveloped, DNA-containing HBV particles/mL each week), and is with minimal equipment easily adaptable to most laboratory settings.

## 1. Introduction

An estimated 2 billion people have been infected with hepatitis B virus (HBV) [1], and more than 257 million patients are suffering from chronic infection [2]. Approximately 20–30% of these patients will develop severe consequences including liver cirrhosis and hepatocellular carcinoma such that every year an estimated 887,000 people die from HBV-related liver diseases [3,4]. Since no reliable curative treatments are available, there remains an urgent need for the discovery and development of new curative therapeutic approaches. To achieve this goal, researchers utilize in vitro and in vivo HBV models that require high-titer and high-purity HBV stocks. However, due to the structure and the replication cycle of HBV, production of these stocks is often associated with expensive equipment or a high amount of expenditure and time.

HBV is a small, enveloped, partially double-stranded DNA virus belonging to the *hepadnaviridae* family. The infectious enveloped HBV particle, referred to as a Dane particle, has a 42 nm spherical structure consisting of a viral nucleocapsid containing the partially double-stranded DNA genome. HBV membrane includes transmembrane glycoproteins which are further divided into small (SHBs), middle (MHBs), and large (LHBs) hepatitis B surface proteins [5]. Besides fully formed virions, HBV-infected cells also secrete non-infectious subviral particles (SVPs) of spherical or filamentous shape. These SVPs do not contain capsids or HBV genomes and can reach up to 10^4^ to 10^6^-fold higher concentrations than Dane particles in the blood of infected individuals [6]. While Dane particles have an LHBs:MHBs:SHBs ratio of 1:1:4 with an LHBs content of ~17%, LHBs content decreases to 10% in filaments and less than 1% in spheres [7]. Interestingly, SVPs may alter in vitro infection in a dose-dependent manner [8,9,10], and while the influence of these SVPs on HBV infection is not completely understood in vivo, there is evidence that they affect the outcome of infection by binding neutralizing antibodies and preventing CD8 T cell responses [11].

HBV replication is highly restricted to hepatocytes, partly due to hepatocyte-specific expression of the HBV entry receptor, sodium-taurocholate co-transporting polypeptide (NTCP) [12,13] and partly because its transcription relies on hepatocyte-specific transcription factors [14,15]. Cellular entry process is initiated by low-affinity attachment to heparan sulfate proteoglycans (HSPGs), followed by a high-affinity binding of NTCP [16,17]. While HSPGs are expressed on common hepatoma cell lines (e.g., HepG2, Huh7, etc.), NTCP is mostly downregulated, necessitating overexpression of human NTCP in these cells prior to infection [4]. Besides these adaptations, HepG2-NTCP cells require more than 100 infectious Dane particles per cell (MOI 100) to achieve only a small percentage (~30%) of infected cells, and since infection rate and MOI are non-linear, high infection doses of 3000 Dane particles per cell lead to less than 85% infected cells [4]. Given these encumbrances, HBV stocks with both stable infectivity and high-titers are required for robust and reproducible in vitro cell culture models.

Immunocompetent in vivo animal models for HBV are limited since apes (*hominidae*) and the northern tree shrew (*tupaia belangeri*) are the only non-transgenic animals which can be infected with HBV, but availability and ethical concerns restrict the use of these animal models. Therefore, current strategies to eliminate chronic HBV infection are being tested in more readily available, immunodeficient mouse models with humanized livers or other new transgenic animal models like rhesus macaques [18,19]. Recently, it has been shown that expression of human NTCP renders rhesus macaques susceptible to HBV and thus allows the establishment of a new immunocompetent non-human primate animal model [20,21]. Although natural hosts such as the chimpanzee develop typical HBV infection after inoculation with a few Dane particles, these alternative HBV animal models require doses of 10^6^–10^9^ Dane particles to obtain a proliferative infection [20,22,23]. As injection volumes are limited and impure inocula can lead to anaphylactic reactions or unwanted immunogenicity, high-titer HBV stocks containing purified virions with low concentrations of SVPs and non-enveloped core particles are essential to infect animal models in vivo.

## 2. Results

### 2.1. Analysis of Different HBV Purification and Concentration Methods

Meeting the growing demand of purified HBV stocks, several protocols for HBV purification have been developed over the last four decades. While early protocols used serum from HBV-infected patients, raising problems of HBV quasi-species, inconsistent titers, or contamination with other human pathogens, the establishment of HBV secreting stable cell lines (e.g., HepAD38, HepG2.2.15) allowed the in vitro production of distinct HBV genotypes, with consistent titers of up to 10^8^ genome equivalents (GE)/mL in the supernatant [24,25,26]. Therefore, several different methods have been developed to concentrate and purify HBV from these supernatants.

#### 2.1.1. Centrifugal Filter Devices (e.g., Centricon Plus-70)

**Figure 1:**
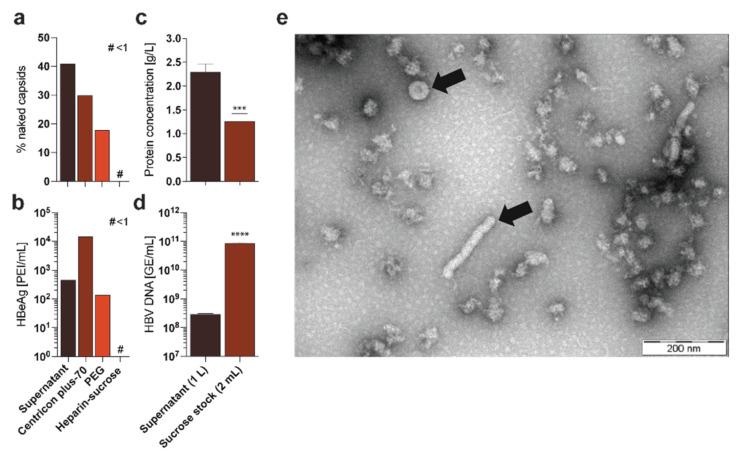
Comparison of different HBV purification methods. HepAD38 cells were cultured and supernatant was purified according to published protocols via Centricon concentration [29], PEG precipitation [30], or the purification protocol described herein: (**a**) CsCl ultracentrifugation followed by a fractionated Dot-Blot analysis was performed to determine the frequency of naked capsids in the different preparations [27]; # < 1; (**b**) HBeAg was quantified via ELISA in the different preparations [4]; # < 1; (**c**) Protein concentration was determined via Bradford assay in supernatant and purified sucrose stock [31]; (**d**) Quantitative PCR was performed to analyze the titer of HBV GE in the supernatant and the purified sucrose stock [4]; (**e**) Electron microscopy was performed after purification of HBV particles via the purification protocol described herein, showing little protein impurity but several virions and filaments (indicated by arrows) [32]. Statistical significance was determined using Student’s *t* test, *** *p* < 0.001, **** *p* ≤ 0.0001.

Cell culture supernatant HBV can be concentrated up to 100-fold with centrifugal filter devices, resulting in a sticky, protein-rich HBV-suspension with a stock concentration of up to 10^10^ GE/mL [27]. However, viscosity of these stocks raises problems in handling, and concentrated SVPs as well as serum proteins hinder addressing specific immunological questions. Furthermore, naked (non-enveloped) capsids secreted by HBV-producing cell lines are also concentrated through this method (Figure 1a) and distort the titration measurements, necessitating more complex methodologies to determine actual titers [27,28]. Most importantly, this method also concentrates the commonly used HBV infection marker HBeAg, which can lead to false-positive results due to input virus and hamper sensitive infection experiments (Figure 1b).

#### 2.1.2. Precipitation Approaches via Chemical Polymers (e.g., Polyethylene Glycol 6000—PEG 6000)

HBV precipitation with PEG works by reducing the solubility of HBV particles in the supernatant by forming PEG-HBV complexes. By centrifugation or ultracentrifugation, these complexes cumulate at the bottom and can be recovered. Although, enrichment of HBV particles is similar as the centrifugal filter devices method, the problem of remaining naked capsids (Figure 1a) and HBeAg still remains (Figure 1b) [24,33]. Moreover, resuspension of PEG-HBV complexes may lead to insoluble precipitates and loss or breakdown of HBV particles during this purification process reducing actual infectivity of the virus stock.

#### 2.1.3. Heparin-Affinity Chromatography

Affinity chromatography of HBV capitalizes on the fact that HBV-LHBs, and to a smaller extent HBV-SHBs, bind to heparin sulfates [34,35]. Thus, HBV-rich supernatant can be loaded onto heparin columns and eluted with an increasing salt-gradient [36]. This process leads to a 100-fold concentration of HBV, whereas more than 95% of the supernatant proteins and most SVPs are removed. However, due to the high-salt concentration in the eluate, additional desalting processes such as dialysis or dilutions are needed for long-term stabilization of the stocks [37]. Indeed, post-process stabilization as well as the complexity and expensive equipment of this purification approach limit the applicability of HBV purification via ÄKTA heparin-affinity chromatography for many HBV research groups.

#### 2.1.4. Sedimentation Centrifugation via Sucrose Gradients

Sedimentation of HBV particles separates Dane particles from other proteins and SVPs based on different sedimentation coefficients during sucrose gradient ultracentrifugation. This separation is dependent on factors like duration, centrifugation speed and variable sucrose concentrations, requiring an elaborate adjustment of these parameters to each other [38]. Overall, the major drawbacks of this technique are the limited ability to concentrate HBV (approx. 10-fold) and the capacity of ultracentrifugation tubes, preventing high-volume HBV purification.

#### 2.1.5. Combination of Heparin-Affinity Chromatography and Sedimentation Centrifugation via Sucrose Gradient

Utilizing the advantages and overcoming the drawbacks of different HBV purification and concentration techniques, our protocol consists of a continuous production of HBV-rich cell culture supernatant followed by a streamlined combination of heparin-affinity chromatography and sucrose gradient ultracentrifugation. While, heparin-affinity chromatography offers concentration and purification of high-titer HBV from large supernatant volumes, subsequent sucrose gradient ultracentrifugation of the eluate leads to further concentration, purification, and an additional buffer exchange for long-term cryopreservation.

Purified HBV-rich 2 mL sucrose fractions contain less than 1.3 g/L protein, while protein concentration of the supernatant is around 2 g/L, leading to a reduction of serum proteins of more than 99% (Figure 1c). Moreover, HBeAg concentration in the purified stock was lower than 1 PEI/mL (reduction of more than 99%), minimizing false positive HBeAg results from input virus compared to other purification methods (Figure 1b). Most importantly, purification led to high-titer HBV-rich 2 mL sucrose fractions with up to 1 × 10^11^ enveloped, DNA-containing HBV particles/mL, depending on the supernatant volumes and viral titers (Figure 1d). Finally, performing electron microscopy on the purified stocks indicate highly purified Dane particles with a ratio of approximately 1:0:2:0 (virions:naked capsids:filaments:spheres), while the supernatant of the HBV producer cell line may contain a ratio of 1:1:1000:100,000 (virions:naked capsids:filaments:spheres) [36], demonstrating an effective separation of Dane particles from SVPs and naked capsid (Figure 1e).

Thus, our protocol leads to a significant reduction in protein concentration while the HBV virion concentration increases 300-fold, yielding in high-titer and high pure HBV stocks of up to 1 × 10^11^ enveloped, DNA-containing HBV particles/mL.

### 2.2. Development of This Protocol

**Figure 2:**
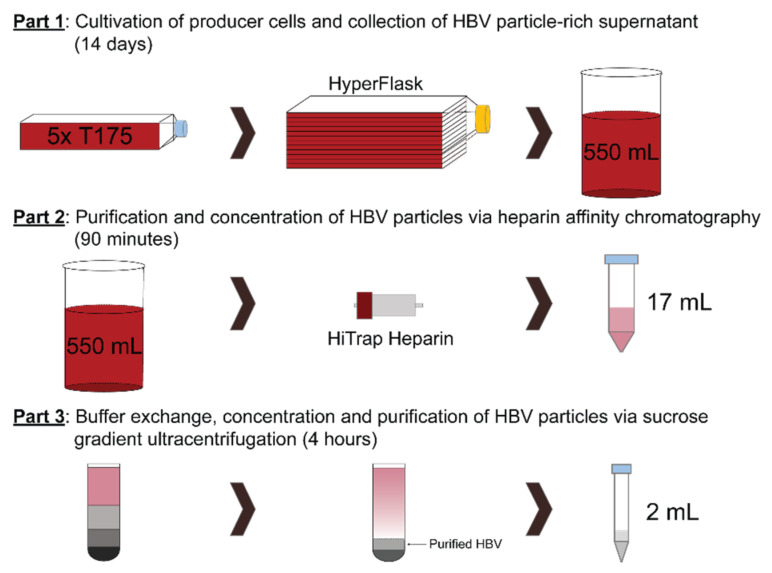
Flow-chart of the protocol. Schematic flow-chart of the protocol, divided into 3 distinct parts. **Part 1**: Cultivation of producer cells and collection of 550 mL HBV particle-rich supernatant per HYPERflask. **Part 2**: Purification and concentration of HBV particles via heparin-affinity chromatography in a 17 mL eluate. **Part 3**: Buffer exchange, concentration and purification of HBV particles via sucrose gradient ultracentrifugation resulting in a 2 mL high-titer HBV fraction.

The overall purification protocol can be divided into three parts (Figure 2).

### 2.3. Overview of the Protocol and Optimization of Each Individual Part

#### 2.3.1. Cultivation of Producer Cells and Collection of HBV Particle-Rich Supernatant

**Figure 3:**
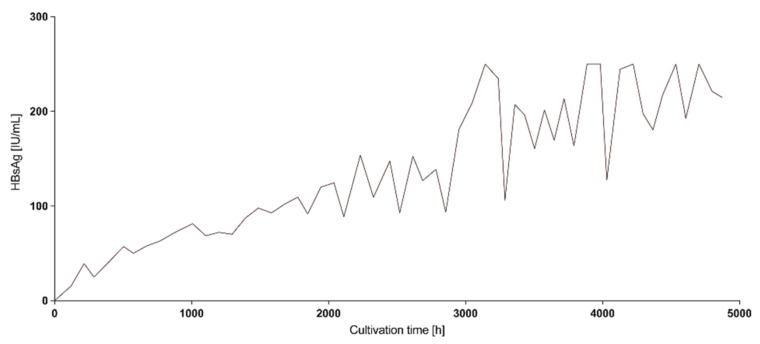
Producer cell lines release more HBsAg over cultivation time. HepAD38 cells were cultivated in a HYPERflask and HBsAg was monitored regularly from the same flask over the course of approx. 5000 h (7 months).

HBV producer cell lines are cultured in a multi-layer flask (e.g., HYPERflask) and HBV particles are secreted into the cell culture supernatant allowing collection of HBV-rich supernatants at regular intervals which can be stored at 4 °C. Interestingly, analysis of HBsAg in the supernatant over time showed that cells can be kept in culture for more than 7 months (~5000 h) without cell splitting while HBsAg concentration in the collected supernatants increased over longer cultivation (Figure 3). Thus, for high-titer stocks, we recommend keeping cells in culture for more than 2 weeks prior to collecting the first supernatant for HBV purification.

#### 2.3.2. Purification and Concentration of HBV Particles via Heparin-Affinity Chromatography

**Figure 4:**
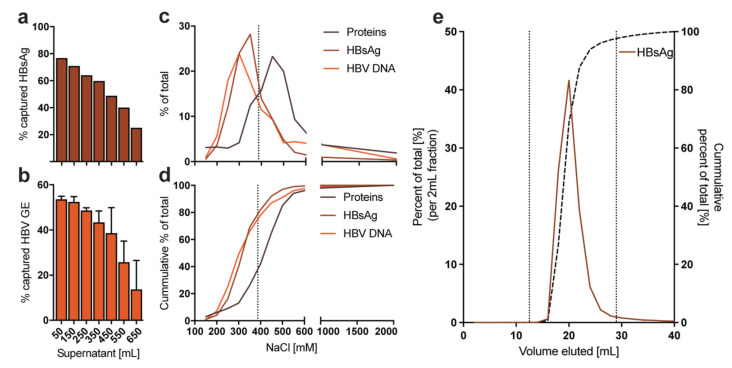
Capacity and optimal elution from 5 mL Heparin HiTrap columns. HepAD38 cells were cultivated in a HYPERflask and different volumes of HBV-rich supernatant were loaded onto a single 5 mL Heparin HiTrap column. Subsequently, fixed elution volumes were analyzed for the percentage of captured (**a**) HBsAg and (**b**) HBV GE compared to the totals present in the respective supernatant loaded. Error bars indicate five technical replicates [4]; (**c**) A single 5 mL Heparin HiTrap column was loaded with 275 mL of HBV-rich supernatant and eluted in 50 mL of elution buffer with different NaCl concentrations. Elution products were analyzed for protein concentration, HBsAg and HBV GE [4,31]. The dotted line indicates the suggested NaCl concentration for elution buffer (approx. 390 nM); (**d**) Similar to panel c, but showing the cumulative percentage of total for each readout; (**e**) Four 5 mL Heparin HiTrap columns were loaded with 550 mL of HBV-rich supernatant in parallel and afterwards eluted in a serial connection. The eluate was fractionated in 2 mL fractions and HBsAg was measured. The dotted lines show the start and end of suggested volume to collect (fraction 12–29 mL).

Collected supernatants are cleared from cell debris through filtration and loaded through a peristaltic pump on heparin columns in a parallel orientation, allowing maximum loading volume of all columns simultaneously. Heparin columns are then connected to the peristaltic pump in series that highly concentrated HBV eluate can be obtained by a single high-salt buffer elution. Interestingly, only approx. 50% of HBV GE in the supernatant are virions binding to the heparin-affinity columns (Figure 4a). The remaining 50% are mostly non-binders or naked capsids secreted by the cells [36]. Capacity of a single HiTrap heparin column is limited by supernatant volume, titer and the ratio of filamentous SVP to HBV virions, such that binding of HBV particles (HBsAg and HBV GE) considerably decreases after 350 mL HBV-rich supernatant per 5 mL Heparin HiTrap column (Figure 4a,b). Thus, we recommend an additional 5 mL Heparin HiTrap column per 350 mL HBV-rich supernatant in parallel connection. Due to different binding affinities of HBV particles, elution starts at concentrations >200 mM NaCl (Figure 4c). In order to elute ~80% of bound HBV but less than 40% of bound proteins (Figure 4d), we recommend using a 390 mM NaCl elution buffer, which can be made by diluting 25 mL of 10× PBS in 75 mL of distilled water. Since each column has a distinct dead volume of approx. 4 mL, we recommend discarding 4 mL eluate for each additional 5 mL HiTrap column and collecting the next 17 mL, where more than 90% of the bound HBsAg is eluted (e.g., using 4 columns, discard 12 mL and collect the following 17 mL) (Figure 4e).

#### 2.3.3. Purification, Buffer Exchange and Concentration of HBV Particles via Sucrose Gradient Ultracentrifugation

**Figure 5:**
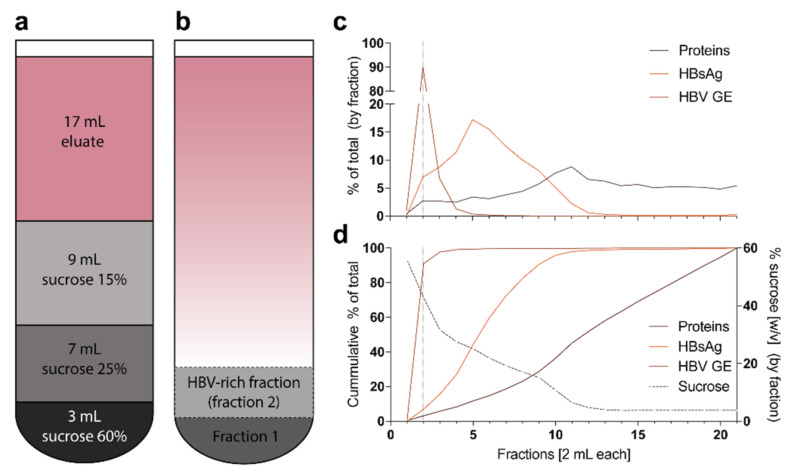
Layering of the sucrose gradient and detailed analysis of the gradient: (**a**) For sucrose gradient ultracentrifugation, the gradient is layered in a SW32Ti tube as displayed; (**b**) After ultracentrifugation, the first 2 mL fraction from the bottom (fraction 1) is discarded and the next 2 mL fraction (fraction 2) of HBV-rich sucrose is collected; (**c**) Heparin-affinity chromatography and sucrose gradient ultracentrifugation were performed as described herein. The gradient was fractionated into 2 mL fractions and each fraction was analyzed for protein concentration via Bradford assay, HBsAg via ELISA and HBV GE via qPCR [4,31]. The dotted line indicates the suggested fraction to collect (fraction 2); (**d**) Similar to panel c, but showing the cumulative percentage of total as well as the percent sucrose concentration (*w*/*v*).

After heparin-affinity chromatography, HBV is unstable in a high-salt buffer and is therefore further purified, buffer exchanged and concentrated via sucrose gradient ultracentrifugation over a three-layer zonal sedimentation gradient (Figure 5a). Gradient is optimized so that the majority of fully-formed Dane particles (approx. 90%) will be in a single 2 mL fraction (Figure 5d), whereas most proteins and SVPs are in upper fractions (Figure 5c,d).

#### 2.3.4. Storage of HBV Stocks

**Figure 6:**
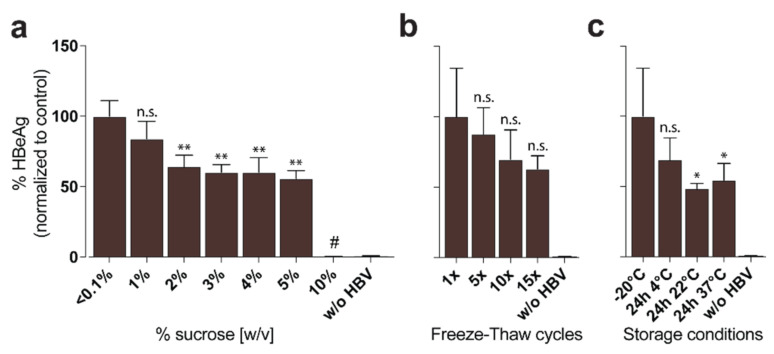
Toxicity and stability of high-titer HBV stocks. HBV was purified with the protocol described herein and aliquoted. HepG2-NTCP were then infected at an MOI of 100 and infection was analyzed by HBeAg ELISA of supernatant 9 days post-infection [4]: (**a**) To examine the inhibiting and toxic effect of sucrose on in vitro infection, sucrose was added to HBV aliquots at different concentrations prior to HepG2-NTCP infection. # = sucrose concentration toxic to cells; (**b**) HBV aliquots were exposed to multiple freeze-thaw cycles prior to HepG2-NTCP infection; (**c**) HBV aliquots were exposed to different storage conditions for 24 h prior to HepG2-NTCP infection. Statistical significance was determined using Student’s *t* test, ** *p* < 0.01, * *p* < 0.05, n.s. not significant.

Since sucrose is non-toxic at low concentrations, high-titer virus stocks containing approx. 42.75% sucrose can be used for high MOI in vitro infections with approx. 5% sucrose final concentration (up to MOI 10,000) (Figure 6a) or in vivo applications [20] and may be frozen without any further processing with high stability at −80 °C for more than 3 years. Multiple freeze-thaw cycles of these stocks (Figure 6b) and short-term storage at room temperature (Figure 6c) are possible, with only a slight reduction of infectivity.

## 3. Materials and Methods

### 3.1. Reagents

#### 3.1.1. HBV-Producer Cell Line Cultivation Medium (~550 mL)

500 mL DMEM/F-12 (Thermo Fisher, cat. no. 11320033)

(If cell line can be selected by Geneticin add 4 mL Geneticin™ selective Antibiotic (G418 Sulfate) (50 mg/mL) (Thermo Fisher, cat. no. 10131027)) 

50 mL Fetal Bovine Serum, certified and heat inactivated (Thermo Fisher, cat. no. 10082139 or US 10082147)

5.5 mL Penicillin-Streptomycin (10,000 U/mL) (Thermo Fisher, cat. no. 15140122)

#### 3.1.2. Elution Buffer (100 mL)

25 mL 10× PBS, pH 7.4 (Thermo Fisher, cat. no. 70011044)

75 mL Distilled Water (Thermo Fisher, cat. no. 15230001)

#### 3.1.3. Wash Solution

50 mL 1× PBS, pH 7.4 (Thermo Fisher, cat. no. 10010023)

*Column wash buffer*:

50 mL 10× PBS, pH 7.4 (Thermo Fisher, cat. no. 70011044)

#### 3.1.4. Heparin Column Storage Buffer (100 mL)

13 mL 10× PBS, pH 7.4 (Thermo Fisher, cat. no. 70011044)

20 mL Ethanol, absolute (200 Proof), Molecular Biology Grade (Fisher Bioreagents, cat. no. BP2818-100)

Distilled Water (Thermo Fisher, cat. no. 15230001) to 100 mL

#### 3.1.5. Sucrose

60% (*w*/*w*) sucrose (Sigma-Aldrich, cat. no. S0389-1KG) in 1× PBS, pH 7.4 (Thermo Fisher, cat. no. 10010023) (filter sterilized) RI: 1.442 nD20

25% (*w*/*w*) sucrose (Sigma-Aldrich, cat. no. S0389-1KG) in 1× PBS, pH 7.4 (Thermo Fisher, cat. no. 10010023) (filter sterilized) RI: 1.372 nD20

15% (*w*/*w*) sucrose (Sigma-Aldrich, cat. no. S0389-1KG) in 1× PBS, pH 7.4 (Thermo Fisher, cat. no. 10010023) (filter sterilized) RI: 1.356 nD20

#### 3.1.6. Cell Culture Solutions

Collagen Solution (550 mL): Collagen R solution 0.2% (Serva, cat. no. 47254.01) 20 mL in distilled Water (Thermo Fisher, cat. no. 15230001) ad 550 mL 

Trypsin Solution (50 mL): Trypsin-EDTA (0.5%), no phenol red (Thermo Fisher, cat. no. 15400054) 5 mL in distilled Water (Thermo Fisher, cat. no. 15230001) ad 50 mL 

500 mL 1× PBS, pH 7.4 (Thermo Fisher, cat. no. 10010023)

### 3.2. Equipment

5× T175 flasks (TPP, cat. no. 90151)

1× Corning^®^ CellBIND^®^ Surface HYPERflask^®^ cell culture vessels (Corning, cat. no. CLS10030)

Masterflex L/S^®^ Digital Drive with Open-Head Sensor (Masterflex, cat. no. HV-07522-28) and Easy-Load^®^ II Pump Head for Precision Tubing (Masterflex, cat. no. HV-77200-30), 600 rpm; 115/230 VAC (Drive and Head: Masterflex, cat. no. HV- 77921-77)

Masterflex L/S^®^ Precision Pump Tubing, Platinum-Cured Silicone, L/S 16; 25 ft (Masterflex, cat. no. HV-96410-16)

Cole-Parmer Male luer with lock ring x 3/16" hose barbs (Cole-Parmer, cat. no. ZM-45518-08)

B. Braun™ Discofix™ 3-Way Stopcock (B. Braun, cat no. 10199361)

Aspirating pipettes, 2 mL, w/o plug (Corning, cat. no. CLS9186-100EA)

HiTrap Heparin HP 5 mL Columns (GE Healthcare, cat. no. 17-0407-03)

Connectors, 1/16″—Luer (GE Healthcare, cat. no. 18-1112-51)

50 mL Conical Centrifuge Tubes (Greiner, cat. no. 227261)

Stericup-HV, 0.45 µm, PVDF, 500 mL, radio-sterilized (Millipore, cat. no. S2HVU05RE)

Prefilter Disks AP15, 75 mm (Millipore, cat. no. AP1507500)

Duran^®^ Original Laboratory Bottle 500 mL (Duran, cat. no. 21 801 44 59)

Duran^®^ Super Duty Beaker (Duran, cat. no. 21 107 54 09)

SW 32 Ti Rotor Package, Swinging Bucket, Titanium, 6 × 38.5 mL, 32,000 rpm, 175,000× *g* (Beckman Coulter Life Sciences, cat. no. 369694)

SW 32 Ti Ultra-Clear Tubes (2 × 50 tubes/box) (Beckman Coulter Life Sciences, cat. no. 331186)

Ultracentrifuge (e.g., Optima L-90K (Beckman Coulter Life Sciences, cat. no. 969349))

Fraction Recovery System, Puncturing (Beckman Coulter Life Sciences, cat. no. 343890)

Thermo Fisher CO_2_ Cell Culture Incubator (Thermo-Fisher, cat. no. 51026406)

HBV high-titer producing cell line (e.g., HepG2.2.15 (ATCC), HepAD38 (generated by C. Seeger, Fox Chase Cancer Center, Philadelphia, PA, USA) or HepG2-pB-HBV1.3). On request, we can provide HepG2-pB-HBV1.3 cell-line (HepG2-based single cell clone cell line, stably transfected with a 1.3-fold HBV genotype D (ayw) and resistance to neomycin antibiotic). CAUTION HBV can cause hepatitis B, a serious liver infection.

### 3.3. Virus Purification via Heparin-Affinity and Sucrose Gradient Ultracentrifugation

#### 3.3.1. Cultivation of Producer Cells and Collection of HBV Particle-Rich Supernatant (Timing 14 Days)

The protocol described herein outlines the production of HBV particles from HBV- or recombinant HBV-expressing cell lines (e.g., HepAD38, HepG2.2.15). An HBV genotype D high-producing cell line (HepG2-pB-HBV1.3) is available upon request.

- Step 1: Proliferate producer cells until they reach confluence on 5 collagen-coated T175 flasks.
- CRITICAL Cultivate all HepG2-based cell lines on collagen-coated plates and flasks. It is sufficient to collagen-coat the flasks by rinsing them with a H_2_O/0.02% Collagen R solution for 5 min followed by aspiration of the remaining Collagen solution.
- Step 2: Collagen coat a 10-layer Corning HYPERflask with 550 mL of H_2_O/0.01%-Collagen R solution and incubate overnight at room temperature. Take care that upper and lower surfaces are equally well coated. Collect H_2_O/0.01%-Collagen R solution and store at 4 °C for future HYPERflask collagen-coatings. Solution can be reused approx. 5 times.
- Step 3: Pre-warm 550 mL of cultivation medium to 37 °C and add 90% of the cells trypsinized off the 5 collagen-coated T175 flasks into the pre-warmed medium bottle. Cultivate the remaining 10% cells again in the 5 collagen-coated T175 flasks for step 6.
- CRITICAL Dispense the cells homogeneously by pipetting them against the wall of the T175 flask.
- Step 4: Pour 550 mL of the warm cell-suspension into the HYPERflask.
  TROUBLESHOOTING
- CRITICAL Remove all remaining air by applying pressure to the center of the HYPERflask, enabling homogenous cell spreading and guaranteeing space for medium extension during 37 °C incubation to avoid a pressure burst of the HYPERflask.
- Step 5: Culture the cells in the HYPERflask for 4 days at 37 °C in a 5% CO_2_ atmosphere to achieve confluence on the upper surfaces of the flask.
- Step 6: Pre-warm 550 mL of cultivation medium to 37 °C, trypsinize the cells regrown in the 5 collagen-coated T175 flasks (step 3) and suspend cells in the pre-warmed medium. Discard medium from the HYPERflask and add 550 mL of cell-suspension to the HYPERflask, flip the flask and cultivate it upside down overnight to allow attachment and growth of cells on the lower surfaces of the flask.
- CRITICAL Dispense the cells homogeneously by pipetting them against the wall of the T175 flask.
- CRITICAL Avoid drying out of cells in the HYPERflask by minimizing time between medium exchange.
- Step 7: Cultivate HYPERflask at 37 °C in a 5% CO_2_ atmosphere and harvest the supernatant in 4 day intervals.
- CRITICAL Discard the first medium collected after each cell plating cycle because it contains a high number of dead and non-attached cells.

TROUBLESHOOTING

CRITICAL As it is known that there is a maturation of HBV particles (t_1/2_ = 4.7 h) [36] and secretion of HBV particles takes place over time, do not harvest supernatant earlier than 2 days after each medium exchange. Depending on the cell density, cells will start detaching and dying after 5–6 days without medium exchange, leading to an optimal exchange interval of 4 days. To facilitate a weekly routine, we recommend exchanging medium in alternating 3 day/4 day intervals.

CRITICAL Store cell culture supernatants at 4 °C overnight prior to heparin-affinity chromatography in order to precipitate serum lipoproteins and cell debris.

CRITICAL Consider: Titer of HBV in the supernatants should be higher than 10^7^ GE/mL to achieve adequate final titers.

PAUSE POINT Generation of HBV-rich supernatant is now completed. Store supernatants at 4 °C but consider that HBV infectivity drops with increasing storage time. For best results, process supernatants after overnight incubation at 4 °C. To facilitate a weekly routine, we recommend purification of two pooled supernatants from the same HYPERflask every 7 days.

#### 3.3.2. Purification and Concentration of HBV Particles via Heparin-Affinity Chromatography (Timing 90 min)

**Figure 7:**
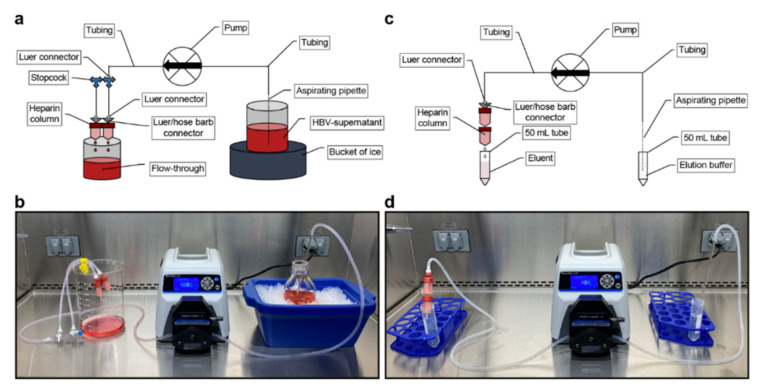
Set-up of the heparin-affinity chromatography apparatus: (**a**) Schematic and (**b**) Pictured set-up for loading of heparin columns. Heparin columns are assembled in parallel connection for loading; (**c**) Schematic and (**d**) Pictured set-up for elution of heparin columns. Heparin columns are assembled in serial connection for elution.

- Step 8: Filter cold, HBV-containing supernatant through a 0.45 µm sterile filter to remove precipitated proteins and remaining cell debris.
  TROUBLESHOOTING
  CRITICAL For best filtration results and saving of filter devices, we recommend pre-filter inlays.
- Step 9: Assemble the purification apparatus without connecting the heparin columns and wash the system with 50 mL of 70% ethanol followed by 50 mL 1× PBS solution (Figure 7a,b).
  TROUBLESHOOTING
  CRITICAL Ensure that the tubing is completely filled with PBS and all remaining air bubbles are removed. Failure to do so will lead to damage of the heparin columns.
- Step 10: Connect 2 heparin columns (HiTrap heparin 5 mL) to the stopcocks in a parallel connection (Figure 7a,b) for every 550 mL of supernatant. To facilitate a weekly routine, we recommend using 4 heparin columns for the weekly volume of 1100 mL.
  TROUBLESHOOTING
- Step 11: Store filtered supernatant on ice and perfuse it through the heparin columns at a flow rate of approx. 10 mL/min per column (e.g., 40 mL/min for 4 heparin columns).
  TROUBLESHOOTING
  CRITICAL Higher loading speed may lower the lifetime of the columns.
- Step 12: After a complete perfusion of the supernatant, remove the columns and flush the system with elution buffer.
  CRITICAL It is important that the columns do not run dry. Ensure that no air is left in the system before you reattach the columns.
  Optional To increase HBV stock purity, but at costs of lower final concentration, columns can be washed with 10 mL of 1× PBS per column.
- Step 13: For elution, assemble columns using a serial connection (Figure 7c,d) and elute with 2 mL/min elution buffer. If you use more than 1 column, discard for each additional column 4 mL of the eluate due to the dead volume and collect the next 17 mL (e.g., using 4 columns, discard 12 mL and collect the following 17 mL (Figure 4e)).
  TROUBLESHOOTING
  CRITICAL Due to variations in dead volume, do not discard any eluate if you elute from a single column.
  CRITICAL Do not exceed the 2 mL/min elution speed in serial connection to avoid damaging the columns and tubing.
  CRITICAL Do not exceed more than 4 columns per elution since they can obstruct during serial elution due to high protein aggregation.
  CRITICAL Do not store the eluent on ice since HBV particles can precipitate due to high salt concentration. Keep eluent at room temperature until step 14.
  PAUSE POINT Purifying and concentrating HBV via heparin-affinity chromatography is now completed and eluent is ready to be further purified, concentrated and buffer exchanged via sucrose gradient ultracentrifugation. We recommend to not exceed storage of eluent for more than 2 h.

#### 3.3.3. Buffer Exchange, Concentration and Purification of HBV Particles via Sucrose Gradient Ultracentrifugation (Timing 4 h)

- Step 14: Carefully overlay sucrose solutions and collected eluate in a SW32Ti ultracentrifugation tube by the following instructions: (from bottom to top) 3 mL sucrose 60%; 7 mL sucrose 25%; 9 mL sucrose 15% and 17 mL eluate (Figure 5a).
- Step 15: Centrifuge the gradient at 175,000× *g* (32,000 rpm) at 10 °C for 3.5 h to perform a separation (Figure 5b).
  TROUBLESHOOTING
  CRITICAL Use fast acceleration and brake.
- Step 16: Fractionate the gradient in 2 mL fractions using a fraction recovery system. The second fraction from the bottom of the gradient is the HBV-rich fraction (Figure 5b).
  TROUBLESHOOTING
  CRITICAL If you do not have access to a fraction recovery system, you may carefully insert a long blunt cannula into the tube and aspirate 2 mL from the bottom. Discard this fraction and aspirate another 2 mL from the bottom to obtain the HBV-rich fraction.
- Step 17: Clean Heparin HiTrap columns by applying 20 mL of 10× PBS solution per column at 5 mL/min in parallel connection. Flush them afterwards with heparin column storage buffer and store them air-tight at 4 °C.
- Step 18: Clean tubings, stop-cocks and fraction recovery system with 70% ethanol to inactivate potentially remaining HBV and store at room temperature.
  Optional Divide HBV stock into smaller aliquots prior to freezing. For in vitro infection experiments, we recommend diluting the sucrose stock 1:1 with fetal bovine serum to lower viscosity and to stabilize HBV particles by covering them with lipoproteins.
  PAUSE POINT Generation of HBV-rich stock is now completed. Stocks should have titers up to 100-times higher than the supernatant (typically higher than 10^10^ GE/mL) and can be stored for more than three years at −80 °C without a notable loss in infectivity. However, we recommend to avoid multiple freeze-thaw cycles and storage in non-frozen stages due to the loss of infectivity (Figure 6b,c).

#### 3.3.4. Troubleshooting

- Overall → Problem: HBV is not infectious → Possible reasons: HBV has been precipitated or denatured → Solution: Check all used buffers. For correct osmolarity, it is crucial to prepare sucrose gradient solutions in 1× PBS. Due to high-salt concentration during elution, it is important to use elution buffer at room temperature and not to store eluate on ice. Thoroughly wash out ethanol-containing buffer with 1× PBS prior to usage of the purification apparatus.
- Step 6 → Problem: Supernatant is cloudy (note: supernatant can be slightly cloudy due to detached cells without affecting the purification process) → Possible reasons: Dead detached cells or bacterial contamination → Solution: Cultivate a 25 mL supernatant-sample in a collagen-coated T175 flask for 4 days and examine it under the microscope.
- → In the case of bacterial contamination (obvious cloudiness and visible bacteria at 40× magnification): start over again from Step 1 and ensure to thoroughly clean spilled medium in the screw thread of the lid after each medium exchange.
- → In the case of dead cells: Check HBV titer (HBsAg or GE/mL) of the supernatant at the next medium exchanges. If titer is further decreasing start over at Step 1. If titer is stable, continue with the protocol and try to avoid to keep too much air in the flask after each medium exchange. Carefully pat out the bubbles in front of the air trap of the HYPERflask. Consider cells are growing on both sides of the layers such that remaining bubbles will lead to punctual drying-out and death of some cells. Additionally, try to minimize time during medium exchange to avoid overall drying-out of the cells.
- → In the case of living cells: Cells probably did not attach adequately to the HYPERflask or have been detached. Check HBV titer in the supernatant of the next medium exchange. If titer has been decreased further start over at Step 1, check collagen solution and increase collagen incubation time in the HYPERflask for up to 2 days. If titer is stable or has been increased, pay attention to a more gentle medium exchange and avoid shaking or friction of the flask.
- Step 6 → Problem: HBV titer is less than 10^6^ GE/mL → Possible reasons: Cells are not dense enough (consider that the bottom layer of the HYPERflask can be examined under the microscope) or do not produce sufficient amounts of HBV → Solution: Keep cells in culture and determine HBV titer at each medium exchange. If titer is not increasing over time until reaching a plateau due to producer cell confluence, use a lower passage of the cell line, escalate antibiotic selection or perform a single-cell selection of a high-producer cell clone or replace the producer cell line (note: a high-producing cell line (HepG2-pB-HBV1.3) is available on request). Check for contamination with other cells, bacteria or mycoplasma.
- Step 10 → Problem: Supernatant is running through parallel heparin columns at different flow-rates (note: slight differences in flow-rate are due to manufacturing and are unproblematic since it will balance during the purification process)→ Possible reasons: One or more columns are blocked or have been used too many times → Solution: Wash blocked column with 50 mL of 10× PBS. If problems remain, exchange the blocked column.
- Step 10 → Problem: Blocking of heparin columns during loading → Possible reasons: Remaining cell debris or proteins plugging the column → Solution: Cooling of the supernatant overnight at 4°C to allow clotting of cell debris and proteins prior to filtering (Step7). Carefully wash blocked column with 50 mL of 10× PBS. If problems remain, exchange it with a new heparin column.
- Step 12 → Problem: Blocking of heparin columns during elution → Possible reasons: HBV concentration is too high and virus is precipitating inside the columns → Solution: Reduce the volume of supernatant applied to each column during loading or elute each column individually. Carefully wash blocked column with 50 mL of 10× PBS. If problems remain, exchange with new heparin columns.
- Step 12 → Problem: HBV is not present in the eluate → Possible reasons: HBV does not bind to the columns or HBV does not elute → Solution: Check HBV titer pre- and post-column. If HBV did not bind to the columns and titers do not differ, exchange the columns. If titers differ, check elution buffer.

#### 3.3.5. Timing

- Step 1–7: Cultivation of producer cells and collection of HBV particle-rich supernatant: 14 days → Production of supernatant every 3 to 4 days for more than 12 months.
- Step 8–13: Purification and concentration of HBV particles via heparin-affinity chromatography: 90 min
- Step 14–18: Buffer exchange, concentration and purification of HBV particles via sucrose gradient ultracentrifugation: 4 h

### 3.4. Virus Purification Using Other Published Protocols

In parallel to the protocol described above, HBV was also purified according to published protocols via Centricon concentration (Centricon Plus-70, Merck cat. no. C3043) [29] and PEG precipitation [30].

### 3.5. Virus Infection

HBV in vitro infection was performed using HepG2-NTCP K7 cells as previously described [4]. Cells were seeded at a density of 50–70% confluence on collagen-coated cell culture plates 48 h prior to HBV infection in Geneticin-free HBV-Producer cell line cultivation medium with 2.5% DMSO. HBV stocks were diluted in Geneticin-free HBV-Producer cell line cultivation medium with 2.5% DMSO and 4% PEG6000. Cells were incubated with that inoculum overnight (maximum of 24 h). Inoculum was removed, cells washed in 1× PBS and further cultivated in Geneticin-free HBV-Producer cell line cultivation medium with 2.5% DMSO.

### 3.6. Quantification of HBV GE via Dot-Blot Analysis

Dot-Blot analysis was performed as previously described [27].

### 3.7. Quantification of HBV GE via qPCR

Quantitative PCR was performed as previously described [4].

### 3.8. Quantification of HBsAg and HBeAg

Quantification of HBsAg and HBeAg was performed using the Abbott Architect platform (Abbott, Ireland, Diagnostic Division).

### 3.9. Protein Quantification

Protein concentration was determined via Bradford assay (Thermo Fisher—cat. no. 23236) [31].

### 3.10. Electron Microscpy

Electron microscopy was performed as previously described [32].

## 4. Discussion

Here we describe a detailed three-step protocol for continuous production of high-titer, high-quality HBV stocks from supernatants of HBV-replicating cell lines. Although many HBV purification and concentration protocols have been published, each of these protocols have different drawbacks and limitations [24,27,28,33,34,35,36,37]. By combining different techniques and fine-tuning each individual step, we simplified and optimized the continuous production of HBV-rich supernatant as well as the entire purification process. Moreover, we minimized necessary equipment and work load, leading to highly purified and concentrated HBV stocks (up to 10^11^ enveloped, DNA-containing HBV particles/mL) (Figure 1c,d).

The HBV purification process via heparin-affinity chromatography and sucrose gradient ultracentrifugation takes less than 6 h, yields in high-titer stocks (up to 1 × 10^11^ enveloped, DNA-containing HBV particles/mL each week), and is with minimal equipment easily adaptable to most laboratory settings as well as attainable for most HBV research groups.

We used this protocol to purify multiple HBV variants and species with different surface proteins including different HBV genotypes and recombinant HBV particles from the supernatant of stable producer cell lines, demonstrating that this protocol is broadly applicable within the HBV field and that these stocks are suitable for both in vivo and in vitro purposes [4,20,38,39,40,41,42].

## Data Availability

Not applicable.
